# Perioperative Complications of Total En Bloc Spondylectomy: Adverse Effects of Preoperative Irradiation

**DOI:** 10.1371/journal.pone.0098797

**Published:** 2014-06-03

**Authors:** Noriaki Yokogawa, Hideki Murakami, Satoru Demura, Satoshi Kato, Katsuhito Yoshioka, Hiroyuki Hayashi, Takayoshi Ishii, Takashi Igarashi, Xiang Fang, Hiroyuki Tsuchiya

**Affiliations:** Department of Orthopedic Surgery, Graduate School of Medical Sciences, Kanazawa University, Kanazawa, Japan; NIH, United States of America

## Abstract

**Background:**

Total en bloc spondylectomy (TES) is associated with a high complication rate because it is technically demanding and involves patients compromised by cancer. Specifically, perioperative complications are more likely to occur in patients receiving preoperative irradiation. We examined the perioperative complications associated with TES in patients receiving preoperative irradiation.

**Methods:**

Seventy-seven patients underwent TES between May 2010 and April 2013. We performed a retrospective review of prospectively collected data for 50 patients with metastatic tumors of the thoracic spine, excluding patients with primary spinal tumors, lumbar spinal metastasis, and combined anterior and posterior approach TES. Patients were divided into 2 groups: those with preoperative irradiation (RT-TES group, 18 patients) and those without preoperative irradiation (TES group, 32 patients). The following perioperative complications, occurring within 2 months of surgery, were compared between the groups: intraoperative dural injuries, epidural hematomas, deep surgical-site infections, postoperative cerebrospinal fluid leakage, wound dehiscence, pleural effusions, and neurological deficits.

**Results:**

Significant differences in patient characteristics were not observed between the RT-TES and TES groups. Perioperative TES complications occurred in 20/50 patients (40.0%). The complication rate in the RT-TES group was 77.8% (14 out of 18), threefold higher than the 18.8% (6 out of 32) in the TES group (*P*<0.01). The incidence of complications, including intraoperative dural injuries, postoperative cerebrospinal fluid leakage, wound dehiscence, and pleural effusions, was significantly higher in the RT-TES group (*P*<0.01).

**Conclusion:**

The perioperative complication rate associated with TES for spinal metastasis was significantly higher among patients receiving preoperative irradiation than among those not receiving preoperative irradiation.

## Introduction

**Figure 1:**
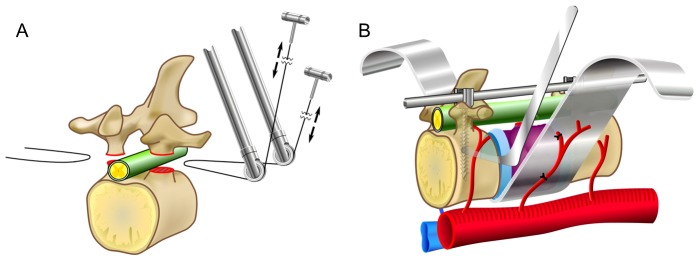
Operative schema for total en bloc spondylectomy. (A) Pediculotomy using a T-saw and en bloc resection of the posterior element; (B) Anterior column osteotomy and removal of the tumor-affected vertebral body.

Total en bloc spondylectomy (TES, Figure 1) is used for complete resection of spinal tumors, including primary malignant, aggressive benign, and metastatic tumors [1], [2]. Intervention with TES reduces local tumor recurrence and improves patient prognosis [3], [4], [5]. However, because of its technically demanding procedures and use in patients who often have complicated medical backgrounds, such as cancer, the rate of perioperative complications is high compared with that associated with other spinal surgeries [6]. Specifically, perioperative complications are more likely to occur in patients receiving preoperative irradiation. To our knowledge, the rate of perioperative complications in TES, with a focus on the adverse effects of preoperative irradiation, has not been previously reported. In the present study, we examined the rate of perioperative complications associated with TES in a single-center, retrospective study.

## Methods and Materials

### Ethics Statements

This study was approved by the ethics committee of Kanazawa University, and written informed consent for the surgery and entry into the research study was obtained from each patient.

### Patient Characteristics

Between May 2010 and June 2013, 77 patients underwent TES, performed by one surgeon in our department. All patients underwent TES for tumor growth that was detected on imaging, unbearable pain, or progression of paralysis with spinal cord compression caused by the tumor. Patients with primary spinal tumors, those who underwent lumbar spine TES, and those who underwent a combined anterior and posterior approach were excluded. We performed a retrospective review of the prospectively collected data for the remaining 50 patients with metastatic tumors of the thoracic spine. The study population included 27 men and 23 women, with a mean age of 57.8 years (range, 24–75 years) at the time of surgery.

### Evaluation Items

Patients were divided into 2 groups: those undergoing TES with preoperative irradiation (RT-TES group, 18 patients) and those not receiving preoperative irradiation (TES group, 32 patients). The following perioperative complications, occurring within 2 months of the surgery, were compared between the groups: intraoperative dural injuries, epidural hematomas, deep surgical-site infections (SSIs), postoperative cerebrospinal fluid (CSF) leakage, wound dehiscence, pleural effusions, and neurological deficits. All cases of accessible intraoperative dural injuries were repaired using a fibrin glue spray; a submuscular negative pressure drainage system was used after surgery, in all cases. In this study, pleural effusion was defined as the required use of a thoracostomy tube.

In the RT-TES group, we also examined the association between each complication and the details of the preoperative irradiation, including total radiation dose and the time interval between the end of the irradiation and the surgery.

### Statistical Analysis

Univariate analysis was performed using Mann–Whitney *U*-tests for continuous variables and Pearson's chi-square or Fisher's exact tests for categorical variables. Statistical significance was set at a *P*-value <0.05. SPSS statistical software version 19 (SPSS, Chicago, IL, USA) was used to perform the statistical analyses.

## Results

Significant differences did not exist between the patients in the RT-TES and TES groups, with regard to patient characteristics (age, sex, body mass index, smoking status, diabetes, American Society of Anesthesiologists grade, Frankel grade, previous spine surgery, preoperative chemotherapy, steroid administration, and serum albumin) or surgical characteristics (operative time, intraoperative bleeding, number of resected vertebrae, and transection of nerve roots) (Table 1). There were no significant differences between the groups with respect to tumor location. The primary tumors were located at the levels of the kidney (5 patients), thyroid (2), breast (2), bladder (2), other organs (6), and at an unknown location in 1 patient in the RT-TES group and at the levels of the kidney (5), thyroid (5), breast (9), stomach (3), other organs (8), and at unknown locations (2) in the TES group. In each patient, a histological examination of the resected tumor confirmed that the lesion was consistent with the primary lesion type.

**Table 1:** Demographic and surgical characteristics of patients.

	RT-TES^a^ group (n = 18)	TES^b^ group (n = 32)	*P*
**Age (y)**	59.7±12.1	56.7±12.7	0.418
**Sex (male:female)**	11∶7	16∶16	0.454
**Body mass index (kg/m^2^)**	23.9±2.8	22.8±3.2	0.120
**Smokers, n (%)**	6 (33.3)	11 (34.4)	0.941
**Diabetes, n (%)**	4 (22.2)	2 (6.3)	0.099
**ASA** c	2.3±0.5	2.1±0.3	0.092
**Frankel grade <D, n (%)**	7 (38.9)	5 (15.6)	0.067
**Previous spine surgery, n (%)**	3 (16.7)	4 (12.5)	0.687
**Preoperative chemotherapy, n (%)**	8 (44.4)	8 (25.0)	0.161
**Administration of steroids, n (%)**	2 (11.1)	2 (6.3)	0.547
**Serum albumin**	3.8±0.4	4±0.4	0.205
**Number of embolized segmental arteries**	3.7±2.1	4.8±1.2	0.081
**Operative time**	467.7±99.8	420.4±84.6	0.102
**Intraoperative bleeding**	683.1±695.7	702.5±1046.3	0.656
**Number of resected vertebrae**	2.1±0.9	1.6±0.8	0.061
**Transection of nerve roots**	3.7±1.9	3.2±1.5	0.359

a RT-TES, patients undergoing total en bloc spondylectomy with preoperative irradiation;

b TES, patients undergoing total en bloc spondylectomy, only;

c ASA, American Society of Anesthesiologists; Numbers in parentheses denotes the percentage.

A total of 37 perioperative TES complications were observed in 20 of the 50 patients (40.0%). Among them, 30 complications were observed in 14/18 patients in the RT-TES group and 7 were observed in 6/32 patients in the TES group. Thus, the rate of complications in the RT-TES group was 77.8% or threefold higher than the 18.8% observed in the TES group. This difference was statistically significant (*P*<0.01); Table 2 shows the details of the complications. Significant differences were noted between the patient groups regarding the numbers of intraoperative dural injuries, postoperative CSF leaks, wound dehiscence, and pleural effusions (*P*<0.01).

**Table 2:** Perioperative complications.

	RT-TES^a^ group (n = 18) n (%)	TES^b^ group (n = 32) n (%)	*P*
**Intraoperative dural injury**	7 (38.9)	1 (3.1)	<0.001*
**Epidural hematoma**	0 (0)	0 (0)	―
**Deep SSI** c	0 (0)	0 (0)	―
**Postoperative CSF** d **leakage**	11 (64.7)	3 (9.4)	<0.001*
**Wound dehiscence**	4 (22.2)	0 (0)	0.006*
**Pleural effusion**	7 (38.9)	2 (6.2)	0.005*
**Neurological deficit**	1 (5.6)	1 (3.1)	0.677
**Total**	14 (77.8)	6 (18.8)	<0.001*

a RT-TES, patients undergoing total en bloc spondylectomy with preoperative irradiation;

b TES, patients undergoing total en bloc spondylectomy, only;

c SSI, surgical site infection;

d CSF, cerebrospinal fluid.

*Statistical significance.

Table 3 shows the influence of the total radiation dose and the time interval between the end of irradiation and surgery for perioperative complications in the RT-TES group. All patients underwent conventional RT, however, in two cases (no. 2 and no. 17), additional irradiation by intensity modulated radiation therapy (IMRT) was performed due to tumor recurrence. In Table 4, the patients were divided into groups depending on the amount of radiation to which each patient was exposed (<40 Gy (median) group and ≥40 Gy group); each complication rate was compared between the groups. Although the difference was not significant, the rate of wound dehiscence was 36.4% in the ≥40 Gy group and 0% in the <40 Gy group. Intraoperative dural injuries, wound dehiscence, and pleural effusions seemed more likely to occur in the ≥40 Gy group. Patients were divided into a <12-month group and a ≥12-month group, according to the time interval between completion of irradiation and surgery (Table 5). The rate of intraoperative dural injuries was 60.0% in the ≥12-month group and 12.5% in the <12-month group (*P* = 0.041). There were no statistically significant differences in the rates of postoperative CSF leakage, wound dehiscence, pleural effusions, or total number of complications.

**Table 3:** Influence of total dose and time interval between the end of irradiation and surgery for perioperative complications in patients undergoing total en bloc spondylectomy with preoperative irradiation (RT-TES).

No.	Age	Sex	Total dose (Gy)	Time interval between irradiation and surgery (months)	Intraoperative dural injury	Postoperative CSF^a^ leakage	Wound dehiscence	Pleural effusion	Neurological deficit
**1**	67	M	41.5	9	-	+	-	-	-
**2**	73	M	55	22	-	-	+	+	-
**3**	68	F	56	39	+	+	-	+	-
**4**	54	F	64	23	+	+	-	+	-
**5**	67	F	30	4	-	+	-	-	-
**6**	42	M	40	36	-	-	-	-	-
**7**	53	F	60	12	-	-	-	-	-
**8**	62	F	50	161	+	+	+	-	-
**9**	49	M	30	25	-	-	-	+	-
**10**	62	M	30	1	-	+	-	-	-
**11**	75	F	36	2	-	-	-	-	-
**12**	65	M	30	8	-	+	-	-	-
**13**	59	F	30	57	+	-	-	-	-
**14**	56	M	40	21	+	+	-	-	-
**15**	26	M	39	2	-	-	-	-	-
**16**	59	M	40	3	-	+	+	+	+
**17**	65	M	82.5	16	+	+	+	+	-
**18**	73	M	50	2	+	+	-	+	-

a CSF, cerebrospinal fluid.

**Table 4:** Complication rates associated with total irradiation dose.

Total dose	<40 Gy (n = 7) n (%)	≥40 Gy (n = 11) n (%)	*P*
**Intraoperative dural injury**	1 (14.3)	6 (54.5)	0.097
**Postoperative CSF** a **leakage**	3 (42.9)	8 (72.7)	0.218
**Wound dehiscence**	0 (0)	4 (36.4)	0.079
**Pleural effusion**	1 (14.3)	6 (54.5)	0.097
**Total**	5 (71.4)	9 (81.8)	0.691

a CSF, cerebrospinal fluid.

**Table 5:** Complication rates associated with the time interval between the end of irradiation and surgery.

Time interval between irradiation and surgery	<12 months (n = 8) n (%)	≥12 months (n = 10) n (%)	*P*
**Intraoperative dural injury**	1 (12.5)	6 (60.0)	0.046*
**Postoperative CSF** a **leakage**	6 (75.0)	5 (50.0)	0.293
**Wound dehiscence**	1 (12.5)	3 (30.0)	0.388
**Pleural effusion**	2 (25.0)	5 (50.0)	0.293
**Total**	6 (75.0)	8 (80.0)	0.805

a CSF, cerebrospinal fluid.

*Statistical significance.

## Discussion

The treatment of patients with cancer has evolved significantly. As a result, patients are surviving longer, and more patients are requiring treatment for spinal involvement. Radiotherapy, a standard treatment for spinal metastasis, is used for local control and pain relief. However, for many patients, local control by radiotherapy alone becomes difficult because of an improved prognosis or inappropriate radiotherapy for solitary spinal metastases of renal or thyroid cancer. In these cases, TES is also considered. Consequently, the opportunities to perform TES on patients who have also received preoperative radiotherapy have increased.

Several studies have shown that irradiation leads to wound complications, such as delayed union, dehiscence, and infection [7], [8]. Ghogawala et al. reported that the rate of major wound complications was 32% in patients who underwent irradiation before surgical decompression for metastatic spinal cord tumors. This rate was threefold higher than that reported for patients not undergoing therapeutic irradiation [9]. The rate of SSIs after surgery for spinal metastasis was also reported to be 31.8% in patients who underwent irradiation, much higher than for those not receiving radiation treatment (1.1%) [10]. Similarly, the rate of en bloc spinal resection complications was 34.3%, with the rate of deep SSI occurrence being higher in patients who had previously undergone irradiation [11]. However, to our knowledge, this study is the first to report the rate of perioperative complications associated with TES, limited to the thoracic level, with a particular focus on the adverse effects of preoperative irradiation.

Poor wound healing is representative of the chronic effects of irradiation resulting from microvascular damage and fibroblast dysfunction [12]. Higher total doses have been previously suggested to enhance the chronic effects [13], as also demonstrated in the current study. We showed that wound dehiscence occurred only in patients receiving ≥40 Gy of preoperative irradiation. In this study, although the difference was not statistically significant, wound dehiscence seemed more likely to occur in patients irradiated ≥12 months before surgery, compared with those receiving irradiation within 12 months of surgery, suggesting that the chronic irradiation effects were involved. Because chronic effects can usually be seen 4–6 months after irradiation, a short delay in post-irradiation surgery is recommended to reduce the potential for wound complications [14]. Conversely, the acute effects of irradiation lead to disorders of vascularization and tissue remodeling due to apoptotic cell death; Devalia et al. suggested that the surgery should be performed 3 weeks after the completion of radiation therapy [15]. Hence, the safest period for performing surgery after a course of radiation therapy is suggested to be 3–6 weeks after irradiation.

Published studies have not reported dural injuries or CSF leakage associated with irradiation. In the present study, most intraoperative dural injuries occurred in patients who had previously undergone preoperative irradiation, and the rate of intraoperative dural injury was significantly higher in patients who had undergone irradiation ≥12 months before surgery, suggesting that the chronic effects of irradiation were responsible for the observed perioperative complications. As the dura mater is mostly composed of collagen fibers, similar to the composition of the skin and subcutaneous tissues, the adverse effects of irradiation are expected to be similar between the dura mater and skin. Although reports have not verified that epidural fibrosis occurs after irradiation, it may occur in a manner similar to that observed in subcutaneous, lung, and liver tissues, where tissue adhesions are often experienced clinically. Epidural fibrosis results in the adhesion of tissue around the dura mater and may cause intraoperative dural injuries, including injuries not recognized during surgery. However, dural injuries must be considered as iatrogenic complications, even though the same surgeon performed all of the TES surgeries. Although most postoperative CSF leakages might be secondary to dural injury, our study also noted that, in patients with relatively acute radiation injuries, postoperative CSF leakage was frequently observed despite the absence of intraoperative dural injury. These observations suggest that acute changes, such as increased permeability or vulnerability of the dura mater, may occur. We are verifying this hypothesis in an experimental animal study.

Surgical invasion of the pleura, accompanying circumferential dissection around the thoracic vertebrae, sometimes results in parietal pleural injury, and is considered to be a major factor in pleural effusion. In some patients, pleural effusion is also induced by the flow of intractable postoperative CSF leakage through a parietal pleural injury. Severe adhesion around vertebrae, secondary to irradiation, may also be one of the causes.

In this study, we did not observe any deep SSIs. In our department, we have used prostaglandin E1 to prevent SSIs in all patients treated by TES since 2006. Recently, we developed a new procedure for the anodization of iodine-containing surfaces that can directly support existing titanium implants. In a basic study using Japanese white rabbits, the results indicated that iodine-supported titanium has antibacterial activity, biocompatibility, and no cytotoxicity [16]. Based on these observations, we have conducted clinical trials in spinal operations using iodine-supported spinal instruments since 2008, and have demonstrated that these instruments have the ability to prevent SSIs, as confirmed in this study [17].

A neurological deficit was observed in 1 patient in each group. Both patients had preoperative neurological deficits (Frankel grade C) due to metastatic spinal cord compression, and although they showed temporary improvement after surgery, their paralysis gradually worsened to Frankel grade A. In the patient without preoperative irradiation, intramedullary metastasis was found surgically, after TES, and this caused the postoperative deterioration. In the patient with preoperative irradiation, in addition to severe preoperative spinal cord compression, there was extensive dissection around the dura mater, requiring the resection of three vertebrae. This necessitated the cutting of six segmental arteries, including two anterior spinal arteries; blood flow was also obstructed because of the irradiation. Together, these occurrences affected postoperative neurological deterioration.

The present study has several limitations, including its retrospective design, small sample size, and differences in cancer types. However, this is the first study to investigate the rate of perioperative complications associated with TES, with a focus on preoperative irradiation. The unique features of this study include the limitation of the study to spinal metastases and the fact that the same type of surgery was performed in all patients; these features separate the present study from other studies reporting the results of spinal tumors en bloc resections. The information provided by this study may contribute to the management of TES, especially in patients undergoing preoperative irradiation.

The results of this study indicate that the perioperative complication rate associated with TES for spinal metastasis is significantly higher in patients receiving preoperative radiotherapy than in those not receiving preoperative radiotherapy. We suggest that careful consideration should be given to patients receiving preoperative irradiation and, whenever possible, preoperative irradiation should be avoided in patients having an indication for TES. However, radiotherapy is widely performed as a standard treatment for spinal metastases making the need for TES inevitable in some patients receiving radiotherapy. Thus, preventive measures to minimize complications need to be established in a future study.
